# Dark papillary muscles sign: a novel prognostic marker for cardiac magnetic resonance

**DOI:** 10.1007/s00330-023-09400-x

**Published:** 2023-01-24

**Authors:** Giovanni Donato Aquaro, Carmelo De Gori, Giulia Grilli, Roberto Licordari, Andrea Barison, Giancarlo Todiere, Umberto Ianni, Matteo Parollo, Crysanthos Grigoratos, Luca Restivo, Antonio De Luca, Lorenzo Faggioni, Dania Cioni, Gianfranco Sinagra, Gianluca Di Bella, Emanuele Neri

**Affiliations:** 1 Academic Radiology Unit, Department of Surgical, Medical and Molecular Pathology and Critical Area, University of Pisa, Via Savi, 10, 56126 Pisa, Italy; 2 University of Trieste, Trieste, Italy; 3 Clinical and Experimental Department of Medicine, University of Messina, Messina, Italy; 4 G. Monasterio CNR-Tuscany Foundation, Pisa, Italy; 5 University of Chieti, Chieti, Italy; 6 Academic Radiology Unit, Department of Translational research and of new technology in medicine and Surgery, University of Pisa, Pisa, Italy

**Keywords:** Papillary muscles, Sudden cardiac death, Cardiac magnetic resonance, Prognosis

## Abstract

**Objectives:**

The prognostic role of left ventricular (LV) papillary muscle abnormalities in patients with preserved LV systolic ejection fraction (LVEF) is unknown. We sought to evaluate the prognosis role of LV papillary muscle abnormalities by CMR in patients with ventricular arrhythmias, preserved LVEF with no cardiac disease.

**Methods:**

A total of 391 patients with > 500/24 h premature ventricular complexes and/or with non-sustained ventricular tachycardia (NSVT), preserved LVEF, and no cardiac disease were enrolled. Different features of LV papillary muscles were considered: supernumerary muscles, papillary thickness, the attachment, late gadolinium enhancement (LGE). Dark-Paps was defined as end-systolic signal hypointensity of both papillary muscles in early post-contrast cine CMR images. Mitral valve prolapse, mitral annular disjunction (MAD), and myocardial LGE were considered.

**Results:**

Dark-Paps was found in 79 (20%) patients and was more frequent in females. It was associated with higher prevalence of mitral valve prolapse and MAD. During a median follow-up of 2534 days, 22 hard cardiac events occurred. At Kaplan-Meier curve analysis, patients with Dark-Paps were at higher risk of events than those without (*p *< 0.0001). Dark-Paps was significantly associated with hard cardiac events in all the multivariate models. Dark-Paps improved prognostic estimation when added to NSVT (*p *= 0.0006), to LGE (*p *= 0.005) and to a model including NSVT+LGE (*p *= 0.014). Dark-Paps allowed a significant net reclassification when added to NSVT (NRI 0.30, *p* = 0.03), to LGE (NRI 0.25, *p* = 0.04), and to NSVT + LGE (NRI 0.32, *p  *= 0.02).

**Conclusions:**

In LV papillary muscles, Dark-Paps is a novel prognostic marker in patients with ventricular arrhythmias and preserved ejection fraction.

**Key Points:**

*• Papillary muscle abnormalities are seen in patients with ventricular arrhythmias and preserved left ventricular ejection fraction.*

*• Early post-contrast hypointensity of papillary muscles in end-systolic cine images (Dark-Paps) is a novel prognostic marker in patients with ventricular arrhythmias and preserved ejection fraction.*

*• Dark-Paps had an additive prognostic role over late gadolinium enhancement and non-sustained ventricular tachycardia.*

**Supplementary Information:**

The online version contains supplementary material available at 10.1007/s00330-023-09400-x.

## Introduction

Papillary muscles have an important role in left ventricular function participating to mitral valve continence during systole [1, 2].

Papillary muscle abnormalities may be associated with different cardiac conditions as congenital heart disease, cardiomyopathies, and ischemic heart disease (papillary ischemia or necrosis) as well as cardiac neoplasm, but they may also be found in otherwise normal heart. [3–5]. Papillary abnormalities range from apparently benign variants as supernumerary muscles, anomalous insertion, thickening of one or both muscles, to overt signs of tissue damage as fibrosis, calcifications, rupture or neoplasm, and eventually to congenital conditions as parachute mitral valve and double chamber left ventricle (LV) [6–9].

Papillary muscles abnormalities may also be associated with mitral valve anomalies elongation of leaflets, prolapse, and maybe also mitral-annular disjunction (MAD). The prognostic role of MAD was assessed in the last few years, and a causative association between MAD and myocardial fibrosis of lateral myocardial wall and the relative greater risk of ventricular arrhythmias was recently described [10–16]. Cardiac magnetic resonance (CMR), with a well-known prognostic role in different non-ischemic cardiac conditions [17, 18], is the ideal imaging technique for the evaluation of papillary muscles, providing information on morphology, function, and tissue characterization [19–21]. In our clinical experience with CMR, we noted the presence of systolic hypointensity of both papillary muscles (Dark-Paps) in cine images acquired early after gadolinium-based contrast injection in a small percentage of patients. A small study recently found a significant association between mitral valve prolapse and Dark-Paps [22].

The aim of the present study was to evaluate the prevalence and the clinical and prognostic impact of papillary muscle abnormalities in patients with frequent premature ventricular complexes (PVC) or with non-sustained ventricular tachycardia (NSVT), with preserved LV systolic function and without previous diagnosis of cardiac disease. Also, the association of papillary muscle abnormalities with mitral valve abnormalities as prolapse and MAD was assessed.

## Methods

In this multicenter study, an initial population of 1461 consecutive patients who underwent CMR from March 2006 to August 2015 with ventricular arrhythmias (> 500 premature ventricular complexes/24 h or NSVT at 24h ECG Holter monitoring) were evaluated in two participating centers. Study protocol was approved by the the institutional internal review board. Inclusion criteria were reported in supplemental data. Patients with frequent PVCs or ventricular bigeminism during examination were treated with an oral antiarrhythmic agent for 3 days before CMR examination in order to optimize ECG trigger and to obtain optimal image acquisition. The final population included 391 patients (supplemental figure 1). A group of 50 age- and sex-matched healthy controls with normal ECG and echocardiography and < 10% 5-year risk of CAD was also enrolled as the control group.

### Cardiac magnetic resonance

All the CMR examinations were performed using a 1.5-T scanner with a dedicated cardiac phased array coil. To evaluate papillary muscle, a set of early post-contrast cine steady-state free precession (SSFP) images was acquired in short-axis views from atrioventricular valve plane to the apex with the following parameters: 8-mm slice thickness, no gap, flip angle of 60°, 30 cardiac phases, NEX 1, FOV 35–40 cm, a matrix of 224 × 224, a 45° flip angle, TR/TE ≈ 2, and a bandwidth of 125 KHz.

A set of long-axis view including 4-, 2-, and 3-chamber views and para-axial views (from diaphragm to the entire outflow tract, 5-mm slice thickness, no gap) were also acquired to evaluate papillary muscle and mitral valve anatomy.

Late gadolinium enhancement (LGE) images were acquired 10 min after administration of a 0.5 molar Gd-based contrast agent with a dosage of 0.2 mmol/kg in the same short-axis and para-axial views. An inversion recovery T1-weighted GRE was used with the following parameters: field-of-view 35 mm, slice thickness 8 mm, no gap between each slice, repetition time 4.6 ms, echo time 1.3, flip angle 20°, matrix 224 × 224, reconstruction matrix 256 × 256, and one excitation. The appropriate inversion time was set using a TI-scout pulse sequence.

### Post-processing

The CMR images were independently analyzed by four expert investigators using a dedicated software (CVi42, Circle Cardiovascular Imaging Inc.).

**Fig. 1:**
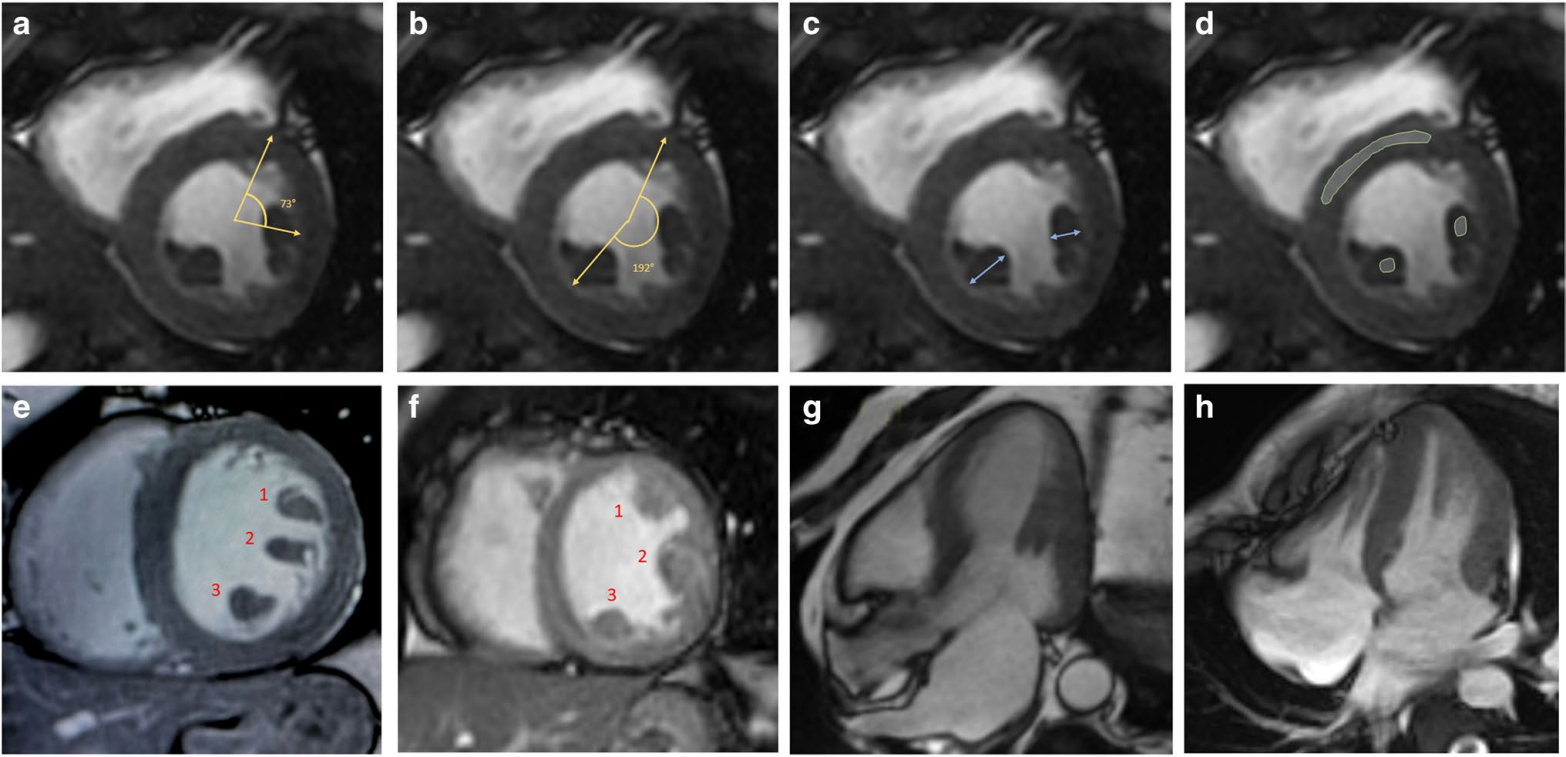
Post processing analysis of anterolateral (AL) and postero-medial (PM) papillary muscle: In the end-systolic cine short-axis images AL and PM papillary muscle radial angles (**a**, **b**) measured as the angle between the anterior right ventricular insertion point and the center of respectively the AL and PM papillary muscle. The end-systolic maximal diameter (**c**) was measured as the maximal transversal diameter of the muscles. In the same early post-contrast end-systolic frames (**d**), the signal intensity of papillary muscles and interventricular septum was measured as shown: signal intensity was measured in regions of interest (ROI) traced in papillary muscles avoiding the blood-pool and in the interventricular, then Dark-Paps was defined when the signal intensity of both papillary muscles was lower than the signal of interventricular septum. In panels **e** and **f**, two examples of supernumerary papillary muscles. A case with normal mid-ventricular papillary attachment and a case of distal attachment are shown respectively in panels **g** and **h**

The characteristics of papillary muscle were assessed in the early post-contrast cine short-axis views. Briefly, the following parameters of the 2 main papillary muscles, namely anterolateral (AL) and posteromedial (PM) muscles, were evaluated (Fig. 1):

1. The presence of supernumerary papillary muscle was evaluated in long- and short-axis views in the presence of an accessory muscle having ≥ 50% main diameter compared with the main papillary muscles and presenting with a separate attachment in the LV walls.
2. The radial angles of the 2 main papillary muscles were measured, in a medio-ventricular short-axis view, as the angle with the ventricular centroid as vertex, one side passing from right ventricular insertion point at the anterior septum and the other side passing in the center of, respectively, the AL and PM papillary muscle.
3. The end-systolic maximal diameter of papillary muscles was manually measured.
4. The signal intensity (SI) of the 2 main papillary muscles and in the interventricular septum was measured in the end-systolic and in the end-diastolic cine frame of a medio-ventricular short-axis view. For each papillary muscle, the end-systolic hypointensity was defined when its measured SI was lower than the SI of the septum [22]. Dark-Paps was defined when both the main papillary muscles had systolic hypointensity. The change of signal intensity from diastole to systole was also measured for each papillary muscle.
5. The type attachment of papillary muscle was visually evaluated and classified as (a) finger-like attachment, presenting as a focal point attachment with few or no trabeculation, or (b) tethered attachment, presenting with a large base of attachment and several trabecular bridges as a ‘mangrove root’ [1, 9].
6. The presence of ectopic papillary muscle attachment was evaluated longitudinally for attachment of papillary muscle in the utmost basal or distal sections of LV or radially for attachment directly from the anterior/anteroseptal or from inferior/inferoseptal wall.
7. The presence of papillary muscle fibrosis was evaluated in LGE images.
8. The morphology and function of mitral valve was also evaluated including the presence of valvular prolapse, mitral annular disjunction (MAD), and regurgitation. Mitral valve prolapse was defined as superior displacement ≥ 2 mm of any part of the mitral leaflet beyond the mitral annulus, according to the American Society of Echocardiography guideline [23, 24]. MAD was defined as an abnormal atrial displacement of the hinge point of the mitral valve away from the ventricular myocardium at the level of the inferolateral wall. Longitudinal MAD distance was measured from the left atrial wall mitral valve leaflet junction to the top of the left ventricular wall during at end systole in all long-axis cine sequences and was defined as present if ≥ 1.0 mm [25].

The presence and distribution of myocardial fibrosis in both the left and right ventricular myocardium was visually evaluated in LGE images.

### Clinical follow-up

Clinical follow-up was performed in all patients for a median of 2534 days (25th–75th 983–3345 days) after the CMR examination. A clinical questionnaire was compiled during periodic ambulatory visits to our institute or via telephone contact. The clinical questionnaire included the definition of the hard cardiac events: cardiac death, resuscitated cardiac arrest, and appropriate implantable cardioverter defibrillator (ICD) shock, sustained ventricular tachycardia (lasting ≥ 30 s at ≥ 100 beats/min). A complete interrogation of the ICD was performed by the referring physician to confirm the appropriateness of the shock. ICD shocks were designated as appropriate if triggered by lethal arrhythmias: ventricular tachycardia above the programmed cutoff of the ICD (12 intervals at > 180 beats/ min) or ventricular fibrillation. The New York Heart Association functional class, data about cardiac hospitalization, and pharmacologic therapy were also collected.

### Statistical analysis

Values are presented as mean ± SD or as median and 25th–75th for variables with normal and non-normal distribution, respectively. Comparison among groups for continuous variables was made with the Student’s *t* test or 1-way analysis of variance or the Kruskal-Wallis test as appropriate. The chi-square test or the Fisher exact test were used, when appropriate, for categorical variables. Analysis of Kaplan-Meier curves was used to evaluate survival free from hard cardiac events among groups. Univariate and stepwise multivariate Cox proportional hazard regression analysis were used to evaluate predictors of hard cardiac events. Variables selected a priori for clinical relevance were first explored with a univariate Cox regression and then different multivariable models were performed. The Harrell-C statistic and McFadden *R*^2^ were calculated for the different models. The incremental value in predicting hard cardiac events by stepwise inclusion was assessed according to the chi-square test by using the Omnibus Tests of Model Coefficients. Reclassification of risk of patients was determined by using net reclassification improvement (NRI) analysis for hard cardiac events. Time-dependent AUC for predicting hard cardiac events was performed using the time-ROC package of R software

## Results

The final population included 391 patients (250 males, 64%) with a mean age of 39 ± 9 years. Characteristics of the patients and healthy control is shown in Table 1. Fifteen (4%) patients had LV dilation and 25 (7%) RV dilation. LGE of LV myocardium was found in 103 (26%) patients, with a non-ischemic in all the cases. A “ring-like” pattern was found in 4 patients (1%). LGE of RV myocardium in was in 21 (5%) of patients, located in the lateral free wall, in the apex, or in the outflow tract.

**Table 1:** Basal characteristics of the whole population

Parameters	Patients	Healthy controls	*p* value
*n*.	391	50	
Age (years)	39 ± 9	37 ± 10	0.18
Males	250 (64%)	36 (72%)	0.35
Weight (kg)	71 ± 14	70 ± 16	0.89
Height (cm)	172 ± 10	172 ± 9	0.9
Family history of sudden cardiac death	11 (3%)	0	0.37
Family history of CAD	26 (7%)	0	0.06
Electrocardiographic abnormalities (negative T Wave)	33 (8%)	0	0.02
PVC > 500/24 h	391 (100%)	0	< 0.0001
PVC/24 h, *n*.	1234 (720–2458)	0	< 0.0001
PVC of LBBB morphology	254 (65%)	0	< 0.0001
PVC of RBBB morphology	70 (18%)	0	< 0.0001
Polymorphic PVC	187 (48%)	0	< 0.0001
Non sustained ventricular tachycardia	34 (9%)	0	0.02
Unexplained syncope	14 (4%)	0	0.37
Therapy:			
Beta-blockers	91 (23%)	0	0.0004
ACE-inhibitors	26 (7%)	0	0.06
Antiarrhythmic drug	25 (6%)	0	0.14
Diuretic	10 (3%)	0	0.62
Cardiac magnetic resonance:			
Left ventricular end-diastolic volume index (mL/m^2^)	82 (72–93)	82 (72–88)	0.44
Left ventricular ejection fraction (%)	64 (59–68)	66 (61–69)	0.11
Left ventricular mass index (g/m^2^)	65 (56–73)	65 (56–72)	0.61
Left ventricular dysfunction	0	0	–
Left ventricular wall motion abnormalities	28 (7%)	0	0.03
Left ventricular dilation, *n* (%)	15 (4%)	0	0.23
Right ventricular end-diastolic volume index (mL/m^2^)	86 (73–96)	86 (77–96)	0.91
Right ventricular ejection fraction (%)	60 (56–66)	63 (68–66)	0.18
Right ventricular ejection fraction < 40%	0	0	–
Right ventricular dilation	25 (7%)	0	0.09
Left ventricular late gadolinium enhancement	103 (26%)	0	< 0.0001
Right ventricular late gadolinium enhancement	21 (5%)	0	0.09
Papillary muscles abnormalities			
Maximal thickness	13 ± 3	13 ± 2	0.91
Systolic hypointensity of both muscles (Dark-PAPs)	79 (20%)	0	0.0002
Systolic AL papillary muscle hypointensity	135 (34%)	3 (6%)	< 0.0001
Systolic PM papillary muscle hypointensity	111 (28%)	2 (4%)	0.0001
Supernumeraries papillary muscles	56 (15%)	8 (16%)	0.99
AL muscle angle (°)	67 (58–78)	63 (53–74)	0.07
PM muscle angle (°)	197 (182–208)	198 (182–208)	0.65
Angle between muscles	128 ± 22	131 ± 21	0.56
Distal or basal attachment	21	0	0.09
Tethered attachment (“mangrove-like”)	88 (23%)	5 (10%)	0.04
LGE papillary muscles	6 (2%)	0	0.99
Prolapse of anterior mitral leaflet	31 (8%)	3 (6%)	0.99
Prolapse of posterior mitral leaflet	18 (5%)	1 (2%)	0.99
Prolapse of both leaflets	10 (3%)	0	0.99
Mitral annular disjunction (MAD)	25 (6%)	0	0.14
Longitudinal MAD distance (mm)	3 (2–4)	0	< 0.0001

Values are mean ± SD or as median and 25th–75th for variables with normal and non-normal distribution

*AL* anterolateral, *PM* posteromedial, *LGE* late gadolinium enhancement, *MAD* mitral-annular disjunction

Supernumerary papillary muscles were found in 56 (15%) of patients, prolapse of the anterior mitral leaflet in 31 (8%), of the posterior in 18 (5%). MAD was found in 25 (6%) of patients and in no healthy controls (overall prevalence 5%). Papillary muscle LGE was identified in 6 (2%) patients. Among healthy controls, no subjects had Dark-Paps despite a minority had systolic hypointensity of a single papillary muscle (6% of AL and 4% of PM muscle). Controls had the same prevalence of supernumerary muscle (16%) than patients.

### Dark-Paps

In cine post-contrast short-axis views, Dark-Paps was found in 79 (20%) patients.

**Fig. 2:**
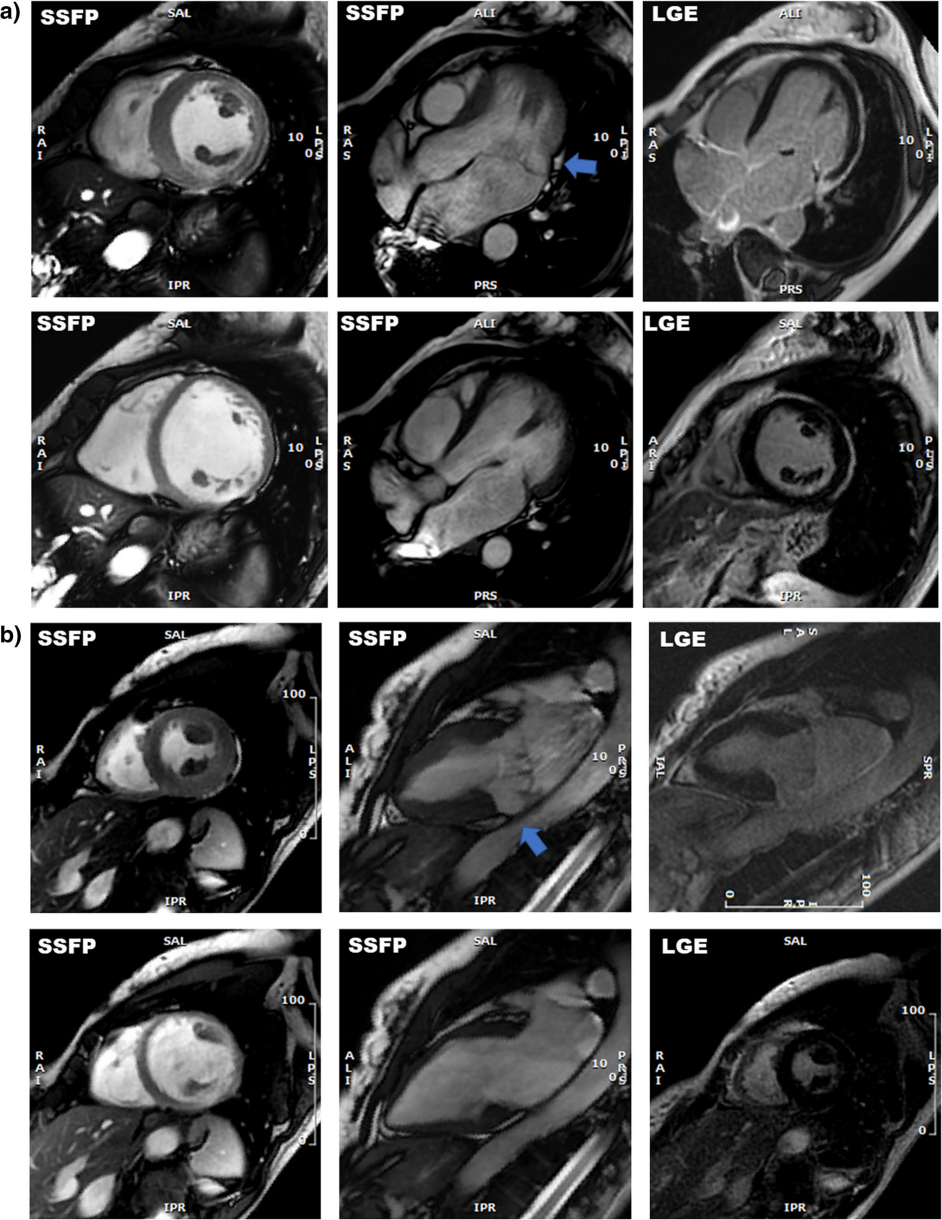
Examples of patients with end-systolic hypointensity of papillary muscle (Dark-Paps) in early post-contrast cine-SSFP: In both case **a** and case **b**, patients had Dark-Paps and mitral annular disjunction with absence of late gadolinium enhancement (LGE)

Characteristics of patients with Dark-Paps is shown in Table 2. Patients with Dark-Paps were older, more frequently females (47% vs 33%, *p *= 0.03), than those without. Patients with Dark-Paps had significantly more frequently mitral prolapse of one or both leaflets. MAD was more frequently seen in patients with Dark-Paps than those without (19% vs 3%, *p *< 0.0001). Dark-Paps was associated with a greater prevalence of tethered, “mangrove-like” attachment of papillary muscles (80% vs 39% in those without, *p *< 0.0001). Moreover, Dark-Paps was associated to a wall thinning in the inferolateral middle wall and to a hyperkinetic inferolateral basal wall. Examples of CMR images of Dark-Paps are shown in Fig. 2.

**Table 2:** Characteristics of patients with or without systolic hypoperfusion of both papillary muscles (Dark-Paps)

	Dark-Paps		
Parameters	Yes (*n *= 79)	No (*n *= 312)	*p* value1
Clinical characteristics:			
Age (years)	46 (25–60)	40 (23–51)	**0.04**
Females	37 (47%)	104 (33%)	**0.03**
Weight (kg)	69 ± 14	71 ± 14	0.29
Height (cm)	171 ± 10	171 ± 10	0.77
Systemic hypertension	4 (6%)	26 (9%)	0.30
Diabetes	0	1	0.98
Dyslipidemia	5 (6%)	17 (5%)	0.97
Family history of sudden cardiac death	3 (4%)	8 (3%)	0.41
Electrocardiographic abnormalities	5 (6%)	28 (9%)	0.49
Non sustained ventricular tachycardia	10 (13%)	24 (8%)	0.16
Unexplained Syncope	3 (4%)	11 (4%)	0.86
Therapy:			
Beta-blockers	23 (29%)	68 (22%)	0.45
ACE-inhibitors	6 (8%)	20 (6%)	0.41
Antiarrhythmic drug	6 (8%)	19 (6%)	0.23
Diuretic	1 (1%)	9 (3%)	0.48
Cardiac magnetic resonance Functional parameters			
Left ventricular end-diastolic volume index (mL/m^2^)	82 (74–91)	82 (72–95)	0.93
Left ventricular ejection fraction (%)	63 (59–67)	64 (59–68)	0.42
Left ventricular mass index (g/m^2^)	63 (56–76)	65 (56–73)	0.77
Left ventricular wall motion abnormalities	6 (8%)	22 (7%)	0.84
Left ventricular dilation	3 (4%)	11 (4%)	0.92
Right ventricular end-diastolic volume index (mL/m^2^)	87 (72–98)	86 (73–96)	0.91
Right ventricular ejection fraction (%)	59 (56–62)	61 (56–66)	**0.01**
Right ventricular dilatation	5 (5%)	20 (8%)	0.37
Tissue abnormalities			
Left ventricular late gadolinium enhancement	26 (33%)	77 (25%)	0.13
Right ventricular late gadolinium enhancement	3 (4%)	18 (6%)	0.69
Papillary muscle abnormalities			
Maximal thickness	13 ± 3	13 ± 3	0.99
Supernumerary papillary muscles	15 (19%)	41 (13%)	0.19
AL muscle angle	67 (60–77)	67 (58–78)	0.89
PM muscle angle	192 (179–205)	197 (183–209)	0.06
Angle between muscles	125 ± 20	129 ± 23	0.14
Distal or basal attachment	5 (6%)	16 (5%)	0.67
Tethered attachment (“mangrove-like”)	60 (80%)	122 (39%)	**< 0.0001**
Hyperkinetic inferolateral basal wall	38 (48%)	42 (13%)	**< 0.0001**
Thinning of inferolateral middle wall	40 (51%)	19 (6%)	**< 0.0001**
LGE of papillary muscles	3 (4%)	3 (1%)	0.09
Prolapse of the anterior mitral leaflet	12 (15%)	19 (6%)	**0.009**
Prolapse of the inferior mitral leaflet	9 (11%)	9 (3%)	**0.003**
Prolapse of both the leaflet	5 (6%)	5 (2%)	0.03
Mitral annular disjunction (MAD)	15 (19%)	10 (3%)	**< 0.0001**

Values are mean ± SD or as median and 25th–75th for variables with normal and non-normal distribution; significant *p* values are marked in bold

*ACE* angiotensin-converting enzyme, *AL* anterolateral, *PM* posteromedial, *LGE* late gadolinium enhancement, *MAD* mitral anular disjunction

### Clinical follow-up

During a median follow-up of 2534 days, 22 hard cardiac events occurred (1 cardiac deaths, 5 resuscitated cardiac arrest, and 11 appropriate ICD shocks, 5 episode of sustained VT). Characteristics of patients with and without hard cardiac events are shown in Table 3. ICD was implanted in 24 patients based on NSVT and clinical evaluation. Hard cardiac events were associated with history of previous NSVT (*p *< 0.0001) and syncope (*p *= 0.004). Among CMR parameters, patients with hard cardiac event had more frequently LV LGE (*p *= 0.004), more frequently Dark-Paps (*p *< 0.0001), supernumerary papillary muscles (*p *= 0.02), and MAD (*p *= 0.001).

**Table 3:** Characteristics of patients with or without cardiac events

	Cardiac Events		
Parameters	Yes (*n *= 22)	No (*n *= 369)	*p* value
Clinical characteristics:			
Age (years)	45 ± 17	39 ± 16	0.09
Males	11 (50%)	239 (65%)	0.16
Weight (kg)	69 ± 12	71 ± 14	0.41
Height(cm)	169 ± 9	172 ± 10	0.19
Systemic hypertension	1 (5%)	29 (9%)	0.68
Diabetes	1 (5%)	0	0.06
Dyslipidaemia	0	22 (6%)	0.61
Family history of sudden cardiac death	0	11 (3%)	0.41
Electrocardiographic abnormalities	4 (20%)	29 (8%)	0.06
Non sustained ventricular tachycardia	6 (30%)	28 (8%)	**< 0.0001**
Unexplained syncope	3 (15%)	11 (3%)	**0.004**
Therapy:			
Beta-blockers	7 (32%)	84 (23%)	0.28
ACE-inhibitors	2 (9%)	24 (7%)	0.30
Antiarrhythmic drug	4 (20%)	21 (6%)	0.02
Diuretic	1 (5%)	9 (3%)	0.35
Cardiac magnetic resonance Functional parameters			
Left ventricular end-diastolic volume index (mL/m^2^)	80 (73–89)	82 (72–95)	0.59
Left ventricular ejection fraction (%)	65 (57–70)	64 (59–68)	0.72
Left ventricular mass index (g/m^2^)	66 (57–87)	66 (57–74)	0.63
Left ventricular wall motion abnormalities	6 (29%)	22 (6%)	**0.002**
Right ventricular end-diastolic volume index (mL/m^2^)	88 (75–98)	87 (73–96)	0.84
Right ventricular ejection fraction (%)	60 (55–66)	61 (56–66)	0.99
Right ventricular dilatation	1 (5%)	24 (7%)	0.61
Right ventricular wall motion abnormalities	11 (50%)	139 (43%)	0.49
Tissue abnormalities			
Left ventricular late gadolinium enhancement	11 (50%)	92 (23%)	**0.009**
Right ventricular late gadolinium enhancement	1 (5%)	20 (6%)	0.78
Mitral valve			
Mitral annular disjunction (MAD)	5 (23%)	20 (5%)	**0.001**
AML prolapse	1 (5%)	30 (8%)	0.54
IML prolapse	1 (5%)	17 (5%)	0.99
Bileaflet prolapse	0	10 (3%)	0.71
Mitral regurgitation (more than mild)	0	2 (1%)	0.93
Papillary muscle abnormalities			
Maximal thickness	13 ± 3	13 ± 3	0.99
DARK-PAPs	14 (64%)	65 (17%)	**< 0.0001**
Supernumerary papillary muscles	7 (32%)	49 (13%)	**0.02**
AL muscle angle	66 (61–74)	67 (57–78)	0.87
PM muscle angle	191 (181–204)	199 (185–210)	0.23
Angle between muscles	126 ± 19	130 ± 23	0.45
LGE of papillary muscles	1 (5%)	5 (1%)	0.29

Values are mean ± SD or as median and 25th-75th for variables with normal and non-normal distribution; significant *p* values are marked in bold

*ACE* angiotensin-converting enzyme, *MAD* mitral annular disjunction, *AML* anterior mitral leaflet, *IML* inferior mitral leaflet, *AL* anterolateral, *PM* posteromedial, *LGE* late gadolinium enhancement

**Fig. 3:**
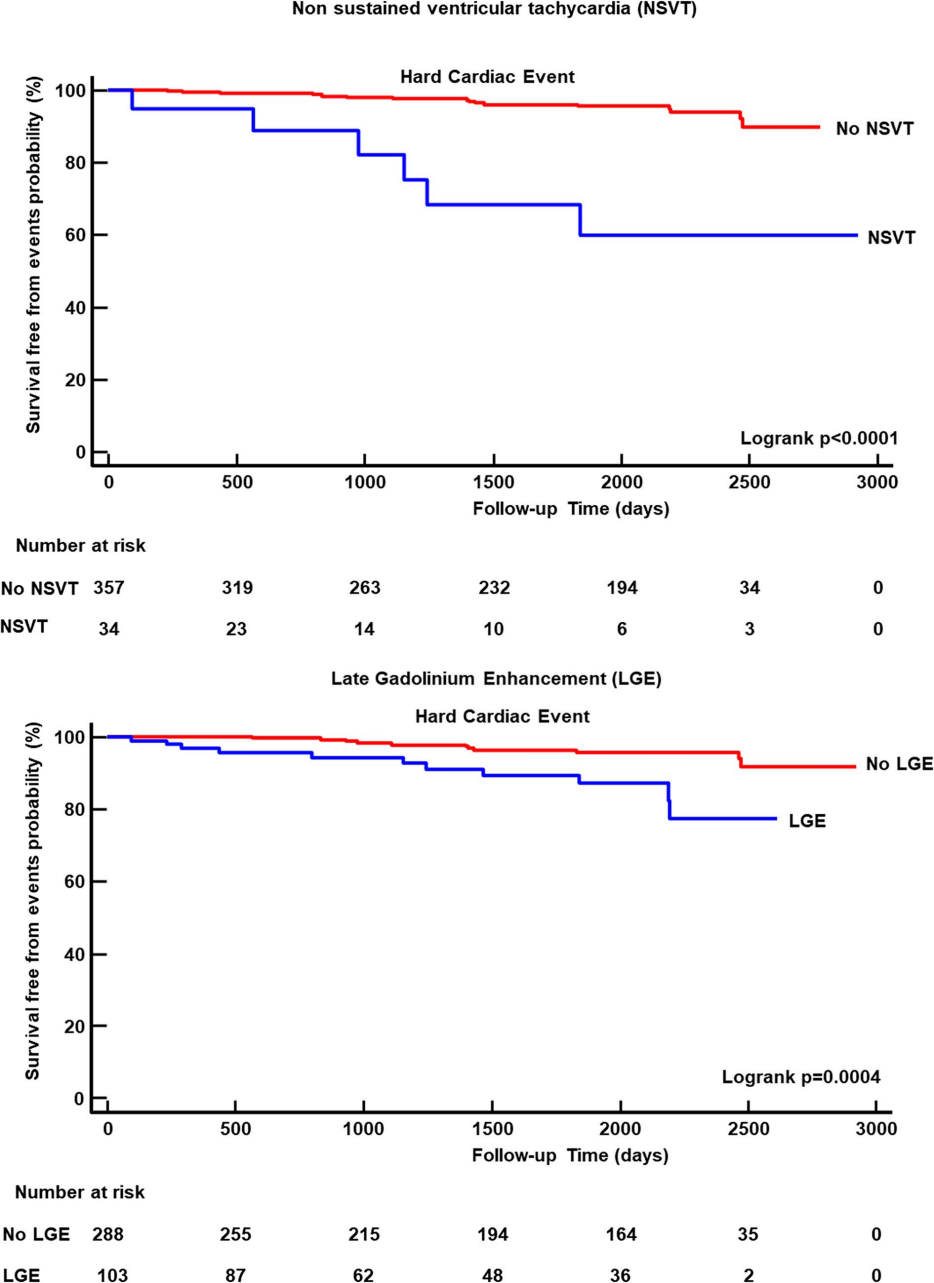
Kaplan-Meier curve analysis for NSVT and LGE

**Fig. 4:**
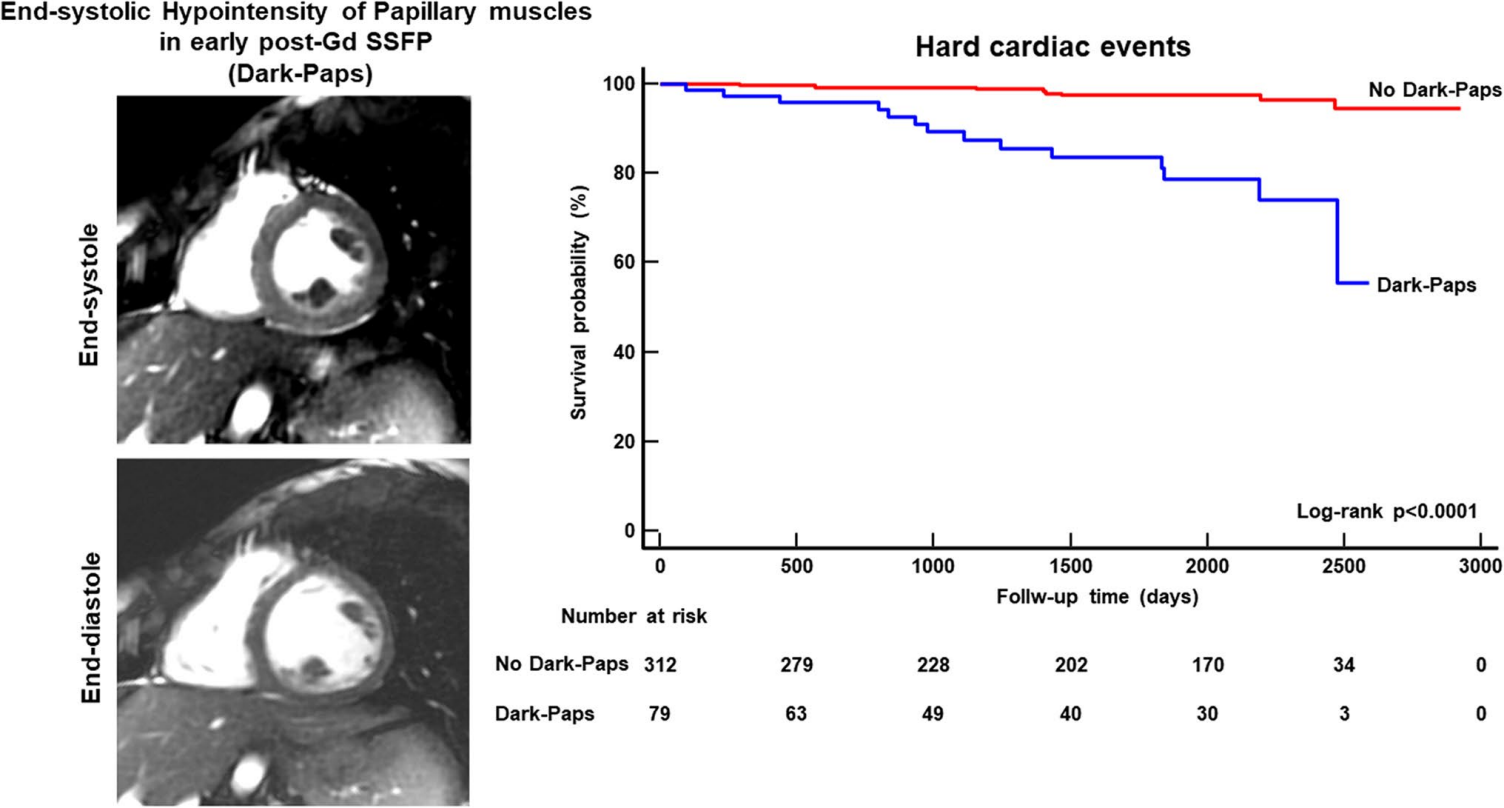
In the left panels, end-systolic (upper panel) and end-diastolic (lower panel) images of a 21-year-old female with Dark-Paps (end-systolic hypointensity of both papillary muscle in early post-contrast cine SSFP short-axis images). This patient was resuscitated from ventricular fibrillation 3 years after CMR. In the right graph, the Kaplan-Meier curve analysis shows that patients with Dark-Paps had worse survival free from hard cardiac events than those without

**Fig. 5:**
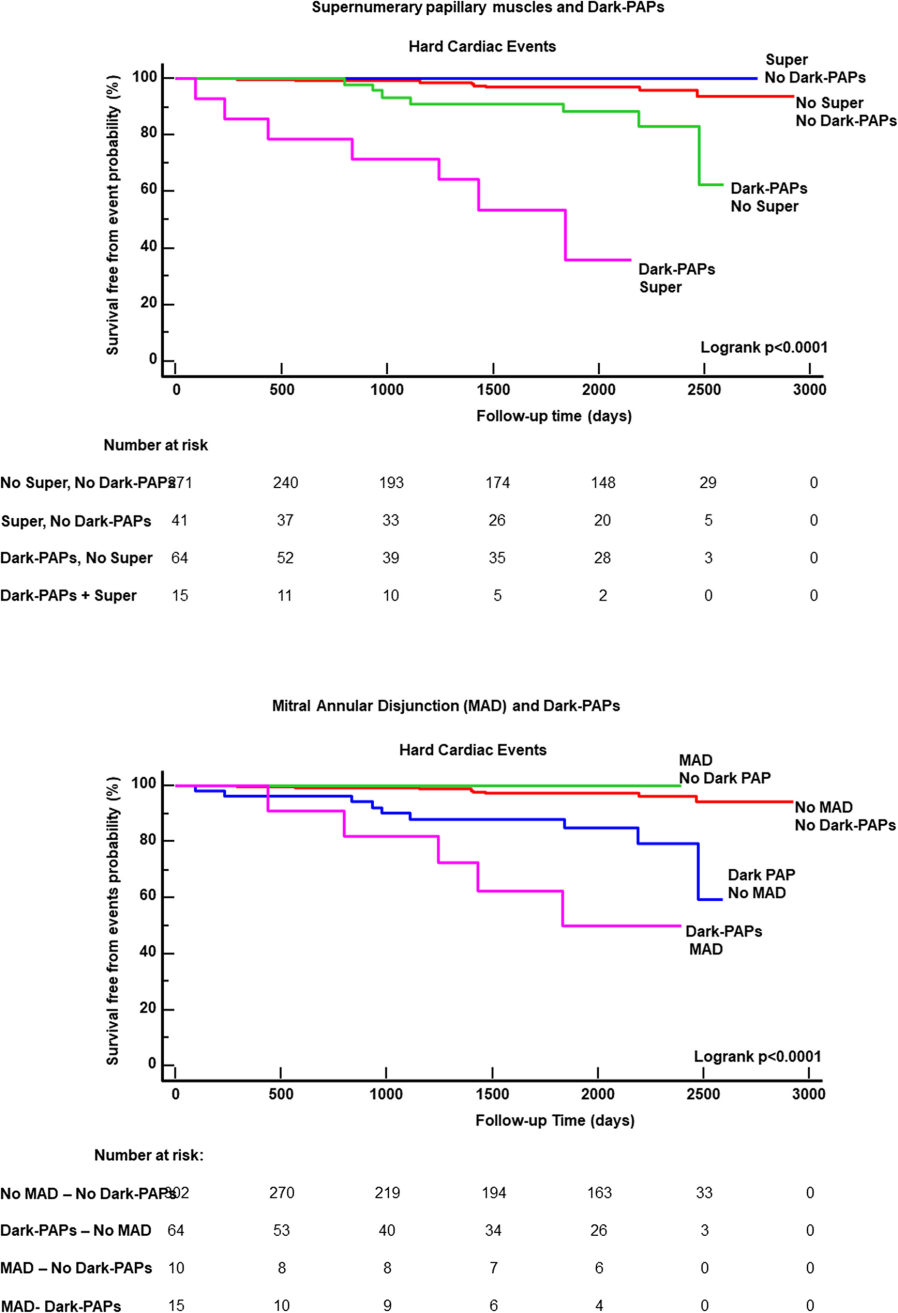
Kaplan-Meier curve analysis for the combination of Dark-Paps with supernumerary papillary muscle (Super) and with mitral annular disjunction (MAD)

As evident in Kaplan-Meier curve analysis of Fig. 4, NSVT(*p *< 0.0001) and LGE (*p *< 0.0001) (Fig. 3) were associated with a greater risk of events. A worse survival-free from events was found in patients with Dark-Paps than those without (Fig. 4, *p *< 0.0001). Supernumerary papillary muscle and MAD were also associated to a worse survival from events. However, as evident in Fig. 5, no event occurred in patients with supernumerary muscle without Dark-Paps as well as in those with MAD without Dark-Paps.

At univariate Cox regression analysis, NSVT, Syncope, LV LGE, supernumerary papillary muscle, MAD, and Dark-Paps were associated with the occurrence of hard cardiac events (Table 4).

**Table 4:** Univariate regression for hard cardiac events

Parameters	HR	CI	*p* value
Clinical characteristics:			
Age	1.02	0.99–1.5	0.1
Premature ventricular complexes > 500/24 h	2.1	0.75–5.9	0.16
Non sustained ventricular tachycardia	6.8	2.6–17.5	**0.0001**
Unexplained syncope	5.2	1.5–17.7	**0.008**
Cardiac magnetic resonance Functional parameters			
Left ventricular end-diastolic volume index (mL/m^2^)	0.99	0.97–1.01	0.52
Left ventricular ejection fraction (%)	0.99	0.95–1.04	0.75
Left ventricular mass index (g/m^2^)	1.00	0.99–1.01	0.46
Left ventricular wall motion abnormalities	8.1	1.5–43	**0.01**
Tissue abnormalities			
Left ventricular late gadolinium enhancement	4.2	1.7–9.8	**0.001**
Right ventricular late gadolinium enhancement	0.68	0.09–5	0.71
Papillary muscles			
Maximal thickness	0.82	0.73–1.03	0.65
Dark-PAPs	8.8	3.7–21.2	**< 0.0001**
Supernumerary papillary muscles	3.1	1.3–7.7	**0.012**
AL muscle angle	1.01	0.98–1.03	0.95
PM muscle angle	0.99	0.97–1.01	0.40
Angle between muscles	0.99	0.97–1.01	0.42
LGE papillary muscles	4.5	0.6–34	0.14
Mitral annular disjunction (MAD)	5.7	2.1–15.7	**0.0008**
Prolapse of the anterior mitral leaflet	0.78	0.10–5.8	0.8
Prolapse of the inferior mitral leaflet	1.6	0.2–11	0.66

Univariate Cox proportional hazards regression analysis was used to evaluate predictors of hard cardiac events; significant *p* values are marked in bold

*AL* anterolateral, *PM* posteromedial, *LGE* late gadolinium enhancement, *MAD* mitral-anular disjunction

Considering the number of events, 4 different models of multivariate Cox regression analysis were performed (Table 5). Every model included Dark-Paps and two other variables with a significant *p* value at univariate. Dark-Paps was significant associated with events in every model.

**Table 5:** Multivariate regression models for predicting hard cardiac events

Parameters	HR	CI	*p* value
Model 1			
Dark-PAPs	7.6	3.1–18.4	**< 0.0001**
Non sustained ventricular tachycardia	4.4	1.6–12	**0.005**
Unexplained syncope	2.4	0.6–9.1	0.2
	*χ*^*2*^ 30.9—*Harrell’s C* 0.79 (0.68–0.90)—*R*^*2*^ 0.26		
Model 2			
Dark-PAPs	8.8	3.6–21	**< 0.0001**
Supernumerary papillary muscles	2.0	0.9–7.4	0.09
Mitral annular disjunction	2.1	0.7–6.4	0.17
	*χ*^*2*^ 28.8—*Harrell’s C* 0.75 (0.63–0.87)—*R*^*2*^ 0.16		
Model 3			
Dark-PAPs	7.0	2.9–17	**< 0.0001**
Left ventricular late gadolinium enhancement	3.5	1.4–8.8	**0.006**
Left ventricular late wall motion abnormalities	2.6	0.9–7.9	0.09
	*χ*^*2*^ 31.9—*Harrell’s C* 0.81 (0.72–0.9)—*R*^*2*^ 0.13		
Model 4			
Dark-PAPs	5.4	2.1–14	**0.0005**
Left ventricular late gadolinium enhancement	2.8	1.1–7	**0.04**
Non sustained ventricular tachycardia	6.5	2.4–17	**0.0002**
	*χ*^*2*^ 35.1—*Harrell’s C* 0.83 (0.75–0.92)—*R*^*2*^ 0.26		

Stepwise multivariate Cox proportional hazards regression analysis was used to evaluate predictors of hard cardiac events; significant *p* values are marked in bold

**Fig. 6:**
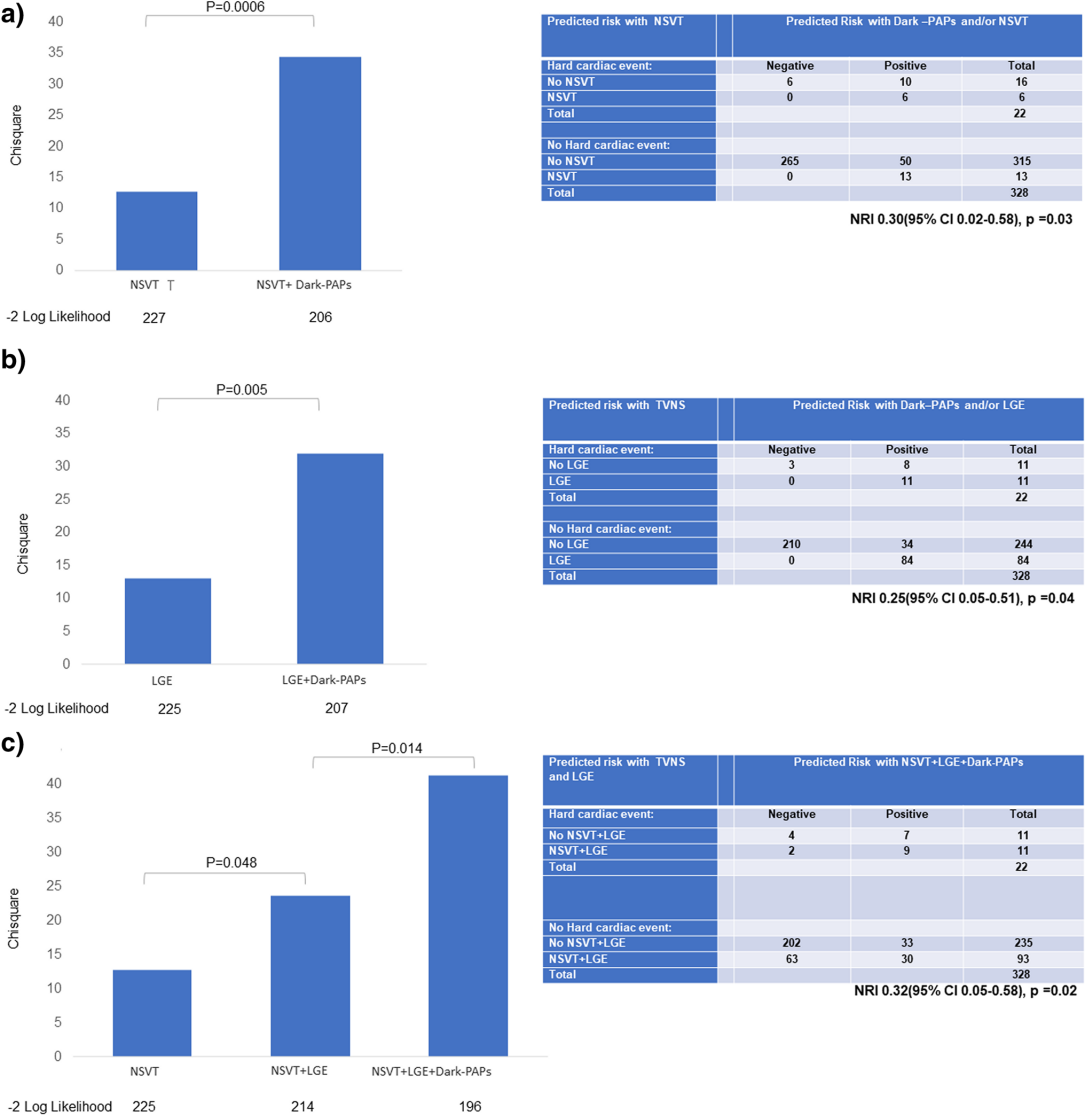
Chi-square improvement and Net Reclassification Improvement analysis

As shown in Fig. 6, Dark-Paps had an incremental value for predicting events, by stepwise inclusion, when added to NSVT (*p *= 0.0006), to LGE (*p *= 0.005), and to the model including NSVT+LGE (*p *= 0.014). As shown in the same figure, Dark-Paps allowed a significant net reclassification when add to NSVT (NRI 0.30, *p* = 0.03), to LGE (NRI 0.25, *p* = 0.04), and to NSVT + LGE (NRI 0.32, *p* = 0.02). Time-dependent AUC for predicting events is shown in supplemental figure 2.

## Discussion

On the best of our knowledge, this was the first study evaluating the prognostic role of papillary muscle abnormalities in patients with ventricular arrhythmias (frequent PVC and/or NSVT) and preserved LV EF and without a definite diagnosis of cardiac disease. We evaluated different aspects of papillary muscles and mitral valve abnormalities, as MAD and mitral valve prolapse. Results of this study may be summarized as follows: (1) in patients with ventricular arrhythmias, Dark-Paps is found in 20% of cases, whereas it was absent in healthy controls; (2) Dark-Paps was an independent predictor of events in all the multivariate models and demonstrated an additive prognostic role over NSVT and myocardial LGE, permitting a significant reclassification of risk.

Papillary muscle abnormalities may play a role in the genesis of ventricular arrhythmias. Indeed, the present study suggests that Dark-Paps might be a new prognostic factor in patients with ventricular arrhythmias and preserved LV EF. Dark-Paps is seen in early post-contrast cine images as well-defined, homogeneous, signal hypointensity of both papillary muscles during the end-systolic phase of cardiac cycle. The explanation of Dark-Paps is not clear, but it is probably caused by a transitory perfusion defect of muscles during the peak of ventricular contraction. Most of papillary muscles do not attach directly to the LV free wall but through the “trabeculae carneae” consisting in a network of muscular strands, acting as tree roots. These trabecaulae may be large, well-defined, and attached in a small focal point of LV wall, giving the aspect of a “finger-like” attachment or, alternatively, they may be thin, multiple, with an ill-defined large attachment, resembling the roots of a mangrove. In our study, 80% of patients with Dark-Paps showed a “mangrove-like” attachment of papillary muscle. This type of attachment could be associated with a rarefaction of blood vessel causing such end-systolic perfusion defect of Dark-Paps.

The mechanical stretch of both papillary muscle and of the inferolateral LV wall caused by MAD and/or by mitral prolapse may participate to this systolic hypoperfusion [26–31]. We considered only MAD of the inferolateral wall that was found 5% of the entire population (patients and controls), accordingly with the prevalence found by Zugwitz et al [32]. However, although the prevalence of MAD and of mitral prolapse was greater in patients with Dark-Paps, 80% of patients with Dark-Paps had neither MAD nor prolapse. The present study confirmed previous evidences of the prognostic role of MAD. However, no event occurred in patients with MAD if Dark-Paps was absent. We cannot exclude that Dark-Paps itself, producing a papillary muscle dysfunction, could be itself cause of MAD or mitral prolapse. As shown in Table 2, 51% of patients with Dark-Paps had a wall thinning of lateral middle wall, whereas it was found in only 5% of other patients. The thin lateral wall as well the “mangrove-like” attachment may suggest a constitutional defect of myocardium, just like a focal “non-compaction,” with a rarefaction of blood vessel of papillary muscles and of lateral middle wall. Further studies are necessary to understand the causes and mechanism of Dark-Paps.

Syncope and NSVT were the only clinical variables associated with hard cardiac event in our population. This result was expected since these parameters were prognostic markers also in cardiac conditions as arrhythmogenic cardiomyopathy, hypertrophic cardiomyopathy, and dilated cardiomyopathy. Moreover, this study confirmed previous evidences, suggesting the prognostic role of LGE in patients with ventricular arrhythmias. However, Dark-Paps demonstrated an additive prognostic role, when added to NSVT, to LGE, and to NSVT + LGE with a significant improvement of predicted risk of events. Based on these results, Dark-Paps may be considered a new marker of structural heart disease associated with a significant increase of risk of malignant arrhythmias in patients with NSVT as well in those with MAD, or with supernumerary papillary muscles. Furthermore, the combination of Dark-Paps and non-ischemic LGE might suggest a more advanced stage of structural heart disease.

The mechanism underlying the arrhythmogenesis associated with Dark-Paps is unknown; however, papillary muscles are vulnerable sites for arrhythmic events. Animal studies demonstrated that papillary muscle may be the site of origin of ventricular arrhythmias that may be treated with radiofrequency ablation [33, 34]. Van Herendael et al found papillary muscles as the site of origin of arrhythmias in 26% of patients with VF or polymorphic ventricular tachycardia [35].

Santoro and coworkers demonstrated that papillary muscles from both ventricles represent an anatomic structure potentially involved in the onset of VF, even in apparently normal structural heart [34]. We found that Dark-Paps appears more often in structurally normal heart and patients with Dark-Paps had not a greater prevalence of LGE than other patients. However, we cannot exclude the possibility of that Dark-Paps could precede the development of fibrosis. It is important to consider that the Dark-Paps was observed in a resting condition. During strenuous physical exercise, the perfusion defect may worsen because of a relative increase of the systole/diastole time ratio, eventually producing a structural myocardial damage with fibrosis or being itself a trigger for arrhythmic events. Future studies with serial CMR or with stress imaging are needed to assess this hypothesis.

Some study limitations have to be mentioned. First, we performed all the CMR using 1.5-T machines and with a high dose of 0.5 molar Gd-based contrast agent (0.2 mmol/kg) and acquired cine-SSFP immediately after injection. Different magnetic field or dosage of Gd-based c.a. could influence the detection of Dark-Paps.

Second, first-pass perfusion imaging could provide further information on myocardial perfusion of lateral wall and even of papillary muscles. However, perfusion imaging is acquired in diastole, with lower spatial resolution than cine-SSFP and usually in only 3 short-axis views. Since Dark-Paps is seen in end-systolic phase, it could have been missed in perfusion images.

Third, T1 and T2 mapping may be used to evaluate papillary muscle tissue abnormalities. However, the spatial resolution of mapping is lower than cine-SSFP and considering the dimension of papillary muscles, the risk of partial volume effect may be relevant with these techniques.

## Conclusions

Among papillary muscles parameters, Dark-Paps emerged as a novel marker for prediction of hard cardiac events in patients with preserved LV EF and ventricular arrhythmias. Dark-Paps has an incremental prognostic role over NSVT and LGE. Dark-Paps is associated with MAD and mitral valve prolapse and may be used to improve risk stratification of long-term arrhythmic event.

## Supplementary Information

ESM 1 (DOCX 926 kb)

## Abbreviations

CMR: Cardiac magnetic resonance
Dark-Paps: Hypointensity of both papillary muscles in end-systolic cine short-axis images acquired early after gadolinium-based contrast injection
ICD: Implantable cardioverter defibrillator
LGE: Late gadolinium enhancement
LV: Left ventricle
MAD: Mitral-annular disjunction
NRI: Net reclassification improvement
NSVT: Non-sustained ventricular tachycardia
PVC: Premature ventricular complexes
RV: Right ventricle

## Declarations

### Guarantor

The scientific guarantor of this publication is Giovanni Donato Aquaro.

### Statistics and biometry

Dr. Giovanni Donato Aquaro provided statistical expertise.

### Methodology

• prospective

• observational

• multicenter study
