# Supplementary material for: Dark papillary muscles sign: a novel prognostic marker for cardiac magnetic resonance
Source: Eur Radiol. 2023 Jan 24;33(7):4621–36. doi: 10.1007/s00330-023-09400-x (PMC10289986; doi:10.1007/s00330-023-09400-x)

**Supplemental figure 1: Flow chart visualizing the derivation of the study population:** PVC= premature ventricular contraction; NSVT= non-sustained ventricular tachycardia; IHD= ischemic heart disease; LV= left ventricle; RV= right ventricle; CMR= cardiac magnetic resonance.

**
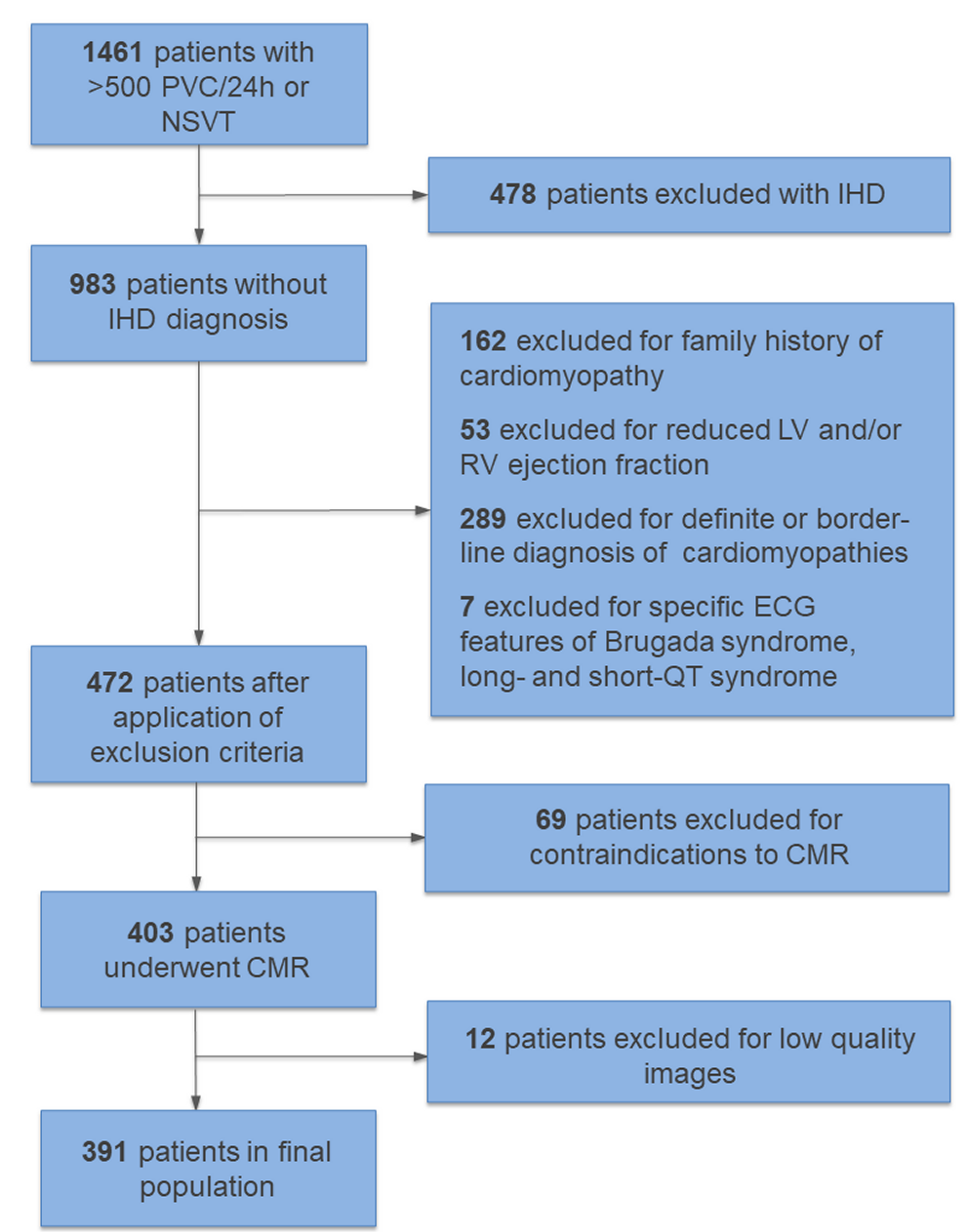
**

**Supplemental figure 2: time dependent AUC curves.** As evident, the time-AUC of Dark-Paps was constantly greater than 0.5 during the first 2000 days of follow-up with a median which was significantly higher than that of NSVT (p<0.0001) and of LGE (0.004). However, the best time-AUC was achieved using the model NSVT + LGE + Dark Paps (AUC 0.89, 95% CI 0.88-0.90).


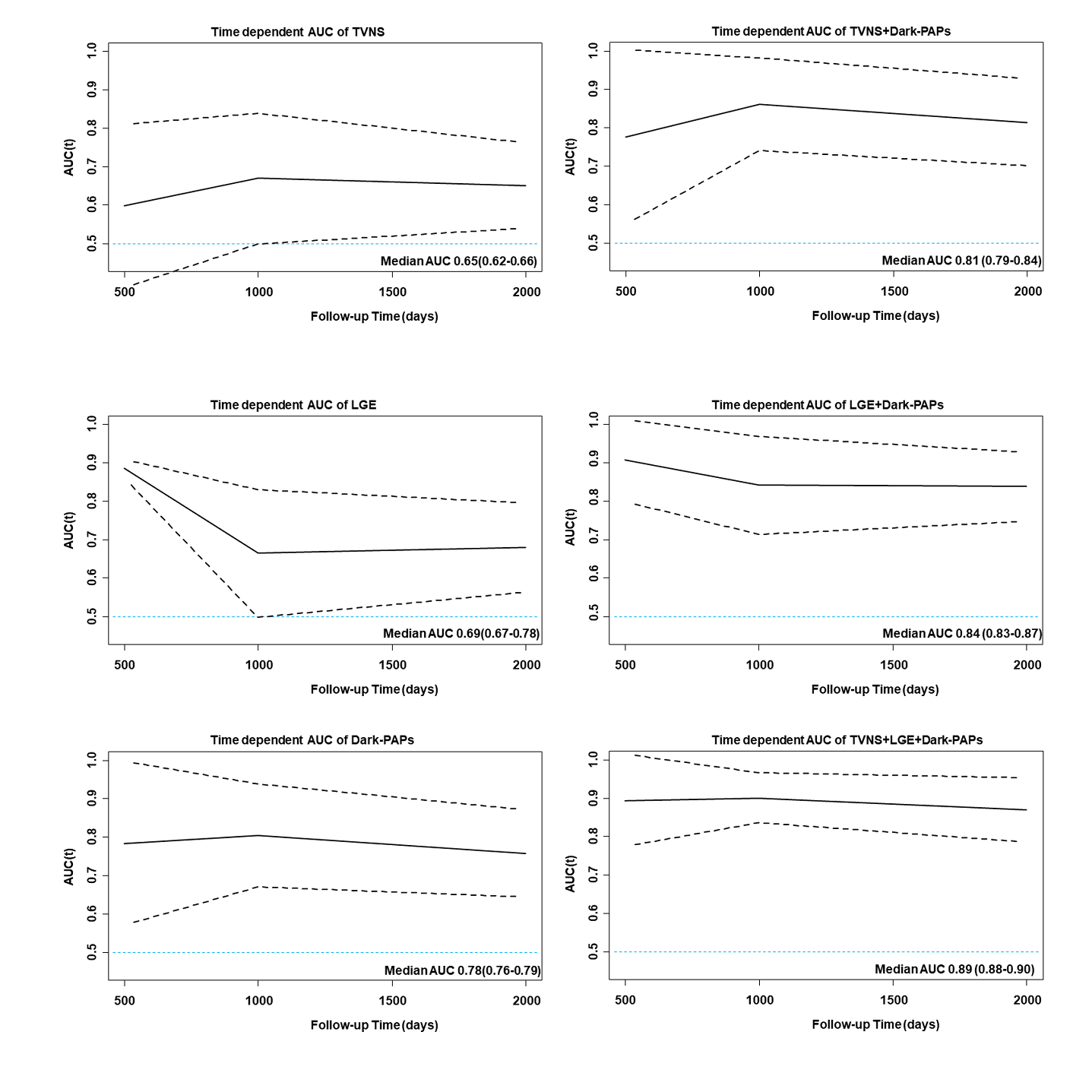

Supplement: Supplementary file 1 — (DOCX 926 kb) [file 330_2023_9400_MOESM1_ESM.docx]
